# Strength Proxies Explain Balance Task Performance by Proximity to Peak Height Velocity in Young Acrobatic Gymnasts

**DOI:** 10.3390/jfmk9040255

**Published:** 2024-12-04

**Authors:** Ignacio Gómez-Dolader, Alejandro Martínez-Cruces, Pureza Leal-del-Ojo, Luis Arturo Gómez-Landero

**Affiliations:** 1 Physical Performance & Sports Research Center, Universidad Pablo de Olavide, Ctra. de Utrera, km. 1, 41013 Seville, Spain; igna.dolader@gmail.com (I.G.-D.); alemc008@gmail.com (A.M.-C.); 2 San Isidoro University Center, Cartuja Island, 41092 Seville, Spain

**Keywords:** center of pressure, stage of maturity, adolescence, postural sway, associations, headstand, acrobatic gymnastics

## Abstract

**Background:** Balance tasks are critical for performance in acrobatic gymnastics, where athletes often train and compete in mixed-age groups with varying maturational stages. To improve individualized training, in this cross-sectional study, the relationship was examined between strength capacity and balance task performance in female gymnasts at two maturational stages based on peak height velocity (PHV). **Methods:** Circa-PHV (n = 17, 11.92 ± 1.7 years) and post-PHV (n = 17, 16.47 ± 1.8 years) participants performed static balance tasks (standing on blocks, tandem stance, headstand) while center of pressure (CoP) excursion was recorded, and a proactive balance task (time to stabilization after landing, TTS). Strength assessments included isometric mid-thigh pull, handgrip, countermovement jump (CMJ), and push-up tests. **Results:** Correlational, regression, and inter-group analyses highlighted differences in strength–balance relationships across groups. Maximal isometric strength and CMJ power were the strongest predictors of static standing balance, with greater predictive strength in the circa-PHV group, underscoring the role of maturation in strength–balance interactions. The results also revealed that strength parameters influenced balance differently depending on the task, suggesting that specific balance types (static–proactive) and tasks (standing–inverted) require distinct strength capacities. **Conclusions:** Strength’s influence on balance varies by maturational stage, emphasizing the need for tailored training programs to enhance balance and optimize performance in young gymnasts.

## 1. Introduction

Traditionally, balance has been shown to be one of the skill-related physical fitness components, but its specific training is also recommended for non-athlete population [1]. The development of this ability in static and dynamic tasks allows people to withstand postural sway challenges or destabilizing stimuli caused by their own movement, the environment or other objects, thus being an influential factor in sports performance [2].

In various gymnastic disciplines, such as acrobatic gymnastics, judges evaluate athletes based on holding time, the complexity of movements, and the quality of execution during specific static positions. These positions may involve group formations, known as pyramids, or individual elements [3]. The ability to maintain such positions is referred to as steady-state static balance [4], a key performance variable, particularly in the ‘balance’ and ‘combined’ routines in acrobatic gymnastics [3].

Center of pressure (CoP) excursion, typically measured using a force platform, is one of the most widely used biomechanical variables and is considered the gold standard for assessing postural control [5,6]. Indirect associations have been identified between CoP excursion and the scores awarded by judges during the execution of static pyramids of varying difficulty [7,8]. Specifically, greater CoP excursions, indicating poorer balance, were associated with lower scores. Moreover, CoP excursion has been shown to differentiate between gymnasts of varying roles and ages in acrobatic gymnastics, both in standard standing positions and in specific inverted positions, such as headstands [6].

Another expression of balance in acrobatic gymnastics is proactive balance, which refers to the anticipation of an expected disturbance [4]. For example, during competition exercises, landings following rehearsed gymnastic and acrobatic jumps are common, and stability during these landings is also evaluated by judges [3]. One variable used to assess this stability is time to stabilization (TTS) [9], which has been suggested to be sport-specific, with gymnasts demonstrating shorter TTS compared to swimmers [10]. Additionally, it has been suggested that task specificity and the age of athletes, influenced by years of jumping experience, may affect post-landing stability [11].

A notable aspect of acrobatic gymnastics is that athletes of varying ages often train and compete together within the same competitive categories, where they are subjected to similar physical and technical preparation demands. These categories include the pre-youth (11–16 years), youth (12–18 years), and junior (13–19 years) age groups [12]. These age groups span different stages of adolescence, which are characterized by varying maturation periods and growth rates that influence postural control, with postural sway generally decreasing as age increases [13]. This influence of biological maturation has been expressed in years from peak height velocity (PHV), observing that in circa-PHV, static and dynamic postural control is lower compared to pre-PHV [14], probably due to the multiple physiological changes during accelerated growth in this phase. This age-related effect has been particularly noted in adolescent athletes during static balance tasks [6,13,15], both in general and specific static balance exercises [6].

Furthermore, the influence of maturation on stability during landings seems less clear. A systematic review with meta-analysis concluded that no definitive conclusions could be drawn regarding the development of postural stability during landings in adolescence [16]. While improved landing mechanics have been suggested to occur with maturation [17], no differences in time to stabilization (TTS) have been observed among gymnasts at different maturation stages [15]. Furthermore, greater variability in sagittal plane kinetics and kinematics during single-leg landings has been reported in older adolescent athletes compared to younger ones [18].

Commented motor actions in acrobatic gymnastics involve various types of strength, including isometric, dynamic, and explosive-elastic strength, all of which are key targets for improvement during training. It is reasonable to expect that different manifestations of strength may influence balance ability, with these effects likely varying based on the gymnast’s maturational stage [19]. However, there is a significant gap in knowledge regarding the specifics of this relationship [20]. Numerous studies have not found a clear connection between strength and balance. In children with ages far from the PHV, no association was found between static balance and the maximum rate of force development of the plantar flexors [21], nor between stable and proactive static balance, measured through the functional reach test (FRT), with the maximum voluntary contraction of the knee extensors and the height of the CMJ [22]. Balance training in children far from PHV showed slight, though not statistically significant, improvements in postural sway, plantar flexor strength, and jump height, likely due to immature postural control and attention deficits [23]. However, Gebel et al. [24] suggested in a review study that balance training in youth and young athletes has potential transfer effects on selected health- and skill-related components of physical fitness as well as on sport skills. Hammami et al. [20] found significant correlations between static and dynamic balance with back extensor strength and jumping ability, which were lower in the pre-PHV group, higher in circa-PHV, and slightly decreased in the post-PHV.

Understanding the optimal timing and appropriate level of intensity for applying strength and/or balance training during the developmental stages of young athletes is crucial for effective programming and performance enhancement [20]. This is particularly important in acrobatic gymnastics, where competition categories consist of heterogeneous age groups [25], and many tasks rely heavily on balance as a key performance factor [25]. However, significant uncertainties remain regarding strength capacity and its relationship to different types of balance [4,21,22,23], in young people with different growth rates [20].

Therefore, the primary objective in this study was to evaluate the associations between strength capacity (power, muscular endurance, and maximal isometric strength) and various balance tasks (static balance standing and inverted, and landing stability) in circa-PHV and post-PHV groups of female gymnasts. Specifically, in this study, the aim was to (1) analyze the differences in these associations between the two groups, and (2) identify the strength parameters that can predict performance in static and proactive tasks in both groups. Based on the current evidence and the study objectives, we hypothesize the following: (H1) there will be an inverse relationship between strength parameters and CoP excursion during static balance tasks; (H2) the associations between strength and balance will vary according to maturational status, with stronger correlations expected in the circa-PHV group; and (H3) strength tests will exhibit greater predictive capacity for balance performance in the circa-PHV group.

## 2. Materials and Methods

### 2.1. Study Design

In this correlational and comparative study, a cross-sectional design was employed. Participants were selected through purposive sampling to address the study’s objectives effectively. The required sample size for achieving adequate statistical power was calculated using G*Power software (version 3.1.9.6; Heinrich-Heine-Universität Düsseldorf, Germany). For a power of 80% at a significance level of α = 0.05, with large correlation coefficients (0.6 > r < 0.7) based on prior research [20], a minimum sample size of 13–19 subjects was required. For comparisons between two independent samples (large effect size, 0.8 > d < 1.0), the required sample size ranged from 17 to 26. For simple linear regression (large effect size, 0.35 > f^2^ < 0.6), a minimum of 16–25 participants were needed.

This study formed part of a broader research project funded by the Universidad Pablo de Olavide (Reference: PPI2201), coordinated by the last author of this work. This research adhered to the ethical principles outlined in the Declaration of Helsinki and was approved by the Ethics Committee for Research with Human Beings of Pablo de Olavide University (Code: 23/3-2). Informed consent was obtained from all participants, and for minors, from their parents or guardians, following a detailed explanation of this study’s objectives and methodology.

### 2.2. Participants

Previous research has highlighted the influence of gender on the development of strength and balance in children [26]. Consequently, only female gymnasts were included in this study. Additional inclusion criteria required participants to have at least three years of experience in acrobatic gymnastics, national-level competition experience, and a minimum training commitment of 12–15 h per week. Exclusion criteria included any injuries or conditions that could impair performance in the tests, as well as failure to meet the inclusion criteria.

From an initial sample of 41 gymnasts, 7 male participants were excluded, resulting in a final sample of 34 female acrobatic gymnasts. Maturation status was determined for all participants based on proximity to PHV [27], and they were subsequently divided into the two groups of circa-PHV (n = 17) and post-PHV (n = 17). Predicted “maturity offset”, defined as the time before or after PHV, is a non-invasive indicator of maturity status, and is provided by formula used [27]. Chronological age at prediction minus offset provides an estimate of age at PHV. Thus, negative values indicate that the participant has not yet reached PHV, while a value of zero signifies that PHV has been reached. Positive values indicate that PHV has been surpassed. Higher absolute values reflecting a greater distance from PHV.

Descriptive characteristics of the sample and the results of the comparative analysis between groups are presented in the Section 3.

### 2.3. Procedures

All measurements were conducted over three separate days. On each day, one or two balance tests (e.g., bilateral standing and drop jump landing) were performed, followed by one or two strength tests (e.g., grip strength and push-ups).

Maximum isometric overall strength was assessed using the isometric mid-thigh pull (IMTP) test, conducted with a force gauge (MUSCLELAB, Stathelle, Norway) operating at a frequency of 1000 Hz, anchored to the ground with height-adjustable chains. A warm-up consisting of bodyweight squats and lunges was performed, followed by two 3 s trials at 50% and 75% of perceived maximal effort, with 60 s of recovery between trials. Participants were instructed to assume a comfortable position replicating the second pull phase of the clean, maintaining an upright torso, with knee flexion of 125–145° and hip flexion of 140–150° [28]. The use of lifting straps was prohibited, although chalk was permitted. After a countdown from three, two measurements were taken, with a two-minute recovery period between them, and verbal encouragement was allowed. From each test, the highest peak force was recorded. If the difference between the two measurements was greater than 250 N, a third measurement was conducted.

The countermovement jump (CMJ) was used to assess lower limb neuromuscular capacity, employing the Optojump system (Microgate, Bolzano, Italy) at a frequency of 1000 Hz. A warm-up consisting of 10 bodyweight squats and 10 lunges was completed prior to testing. Gymnasts performed three familiarization attempts to minimize methodological errors, followed by three test measurements [19], with two minutes of recovery between each trial. The parameters obtained from each jump were the height reached and power.

The isometric hand grip strength test was conducted using a CAMRY EH101 dynamometer (General ASDE, Valencia, Spain). The dynamometer was adjusted for each participant, ensuring alignment of the gripping area with the second phalanx. The test was performed with participants standing, maintaining full extension of the shoulder and elbow [19]. Three attempts were made using the dominant and non-dominant hand, each initiated after a countdown from three, and the grip was held for three seconds. A two-minute recovery period was provided between attempts. The maximum force shown in each attempt was the recorded measurement.

Muscular endurance was evaluated using the maximum push-ups test, following the revised protocol by Baumgartner et al. [29]. The warm-up included two sets of ten repetitions with one minute of rest, with participants choosing to support their knees or not, based on perceived effort. For the test, participants assumed a prone position, supporting only their hands and feet on the floor. A single attempt was conducted, in which participants were required to complete full-range push-ups with the full body extended, touching a foam block placed on the floor. The test continued until failure or until the participant failed to touch the foam on two consecutive repetitions, finally recording the total number of push-ups performed.

**Figure 1:**
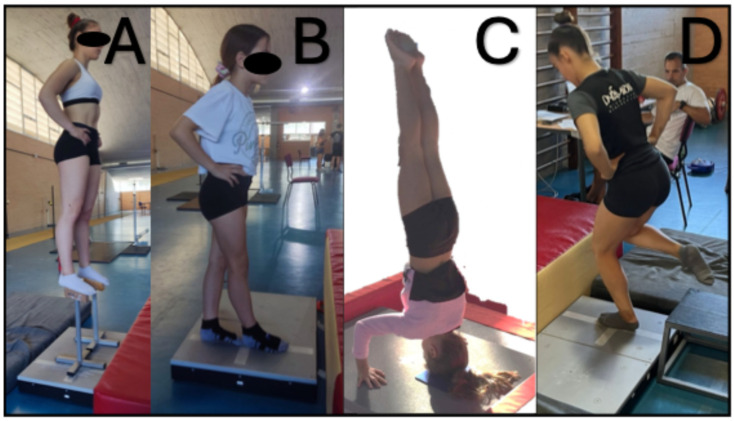
Balance test: bipedal on blocks (**A**), tandem (**B**), headstand (**C**), and landing on single leg (**D**).

Static steady-state balance was assessed in three different postures using a force platform (Sensix^®^, Poitiers, France) at a sampling frequency of 500 Hz to measure CoP excursion. Data collection began once the subject achieved a stable position. After two familiarization trials, three valid attempts were performed, with two minutes of rest between each. The total length travelled in the anteroposterior and mediolateral axes (AP and ML) and the mean speed (SP) were the CoP parameters recorded in each trial. Two tests were conducted in a bipedal stance (i.e., generic), where participants maintained the position for 30 s with hands on hips, gazing at a fixed point ahead. One test involved a full tandem stance, with dominant foot positioned in front (Figure 1B). The second test involved a bipedal stance on acrobatic blocks (bipedal), which was used as an alternative to the standard bipedal stance to further differentiate between gymnasts [30,31]. These acrobatic blocks are standard equipment in acrobatic gymnastics (Figure 1A). Third position was an inverted balance test, specifically a headstand holding 10 s [6], with a pad (made of lesser density rubber composites with fabric bonded to the upper surface) placed on the force plate for the comfort of the head. This element is commonly used in acrobatic gymnastics, where the gymnast maintains full body extension while supporting themselves with three points of contact—head and hands in a triangular formation, with bent elbows (Figure 1C). An assistant monitored each attempt to ensure safety, but did not intervene during the execution.

Proactive balance was evaluated by measuring the time to stabilize after landing on a single leg (TTS) [9]. A force platform, sampling at 1000 Hz, recorded the participants’ body weight in Newtons. Participants, barefoot, stood on a 30 cm high box and stepped forward with hands on their hips, falling onto one leg on a 10 cm high platform, resulting in a 20 cm jump distance (Figure 1D). They had to maintain the landing position for at least 7 s. The trial was repeated if hands moved from the hips, the opposite leg touched the ground, or additional hops were needed for stabilization. After five familiarization trials, three attempts with each leg were recorded, with a 30 s rest between trials.

During the proofreading of the final manuscript, the Writefull tool was employed to refine expressions and correct general spelling. Google Scholar and ChatGPT were consulted to identify specific expressions and concepts, but not to generate or create content. Most suggestions from these tools were not adopted; instead, the terminology and expressions most used in the scientific literature (e.g., circa-PHV) were ultimately selected.

### 2.4. Data Analysis

Depending on the consistency of the measurements, always conducted by the same evaluator for each test, the best value or the average between them was selected based on the intraclass correlation coefficient (ICC type 3,1) with a 95% confidence interval (CI). For the IMTP test, the average peak force was used (ICC = 0.918, 95% CI: 0.862–0.953). In the CMJ, both jump height and flight time were recorded, with the best attempt considered for analysis (ICC = 0.960, 95% CI: 0.934–0.977). Additionally, peak power was included and calculated using the formula: CMJ_Power_ (W) = (51.9 × jump height (cm)) + (48.9 × weight (kg)) − 2007 [32]. For the handgrip test, the average of the best result for each hand was calculated (ICC = 0.954, 95% CI: 0.926–0.971).

In the individual balance tests, the total CoP excursion in both the anteroposterior (AP) and mediolateral (ML) directions, along with the mean CoP speed (SP), were obtained using MATLAB software (v.R2023A). The final value was the average of all attempts. According to the differences in the heights of the sample studied, measurements were normalized to the height of gymnasts [15,33]. ICC values were recorded for bipedal (0.927 ≥ ICC ≤ 0.983, 0.877 ≥ CI ≤ 0.990), tandem (0.990 ≥ ICC ≤ 0.991, 0.983 ≥ CI ≤ 0.995), and headstand tests (0.976 ≥ ICC ≤ 0.983, 0.959 ≥ CI ≤ 0.990).

For single-leg landings, stability was defined as the point when the vertical ground reaction force reached and stayed within 5% of body weight for one second. Initial contact was identified as the point when vertical ground reaction force first crossed 10 N, and SL-TTS was calculated by subtracting this from the onset of stability [9]. The final values were averaged across both legs (ICC = 0.656, 95% CI: 0.427–0.814).

### 2.5. Statistics Analysis

The data analysis was conducted using JASP Statistics Software (v.0.18.0) and Microsoft Excel (2019). Internal consistency reliability of the variables was evaluated with the ICC, with a threshold of ≥0.75 indicating good-to-excellent reliability [34]. The level of statistical significance was set at α = 0.05.

Descriptive statistics, including means and standard deviations, were calculated for all variables. The Shapiro–Wilk test was employed to assess the normality of data distributions. Independent samples *t*-tests were used to compare variables between groups (circa-PHV vs. post-PHV). For variables with non-normal distributions, the Mann–Whitney U test was applied.

Pearson’s correlation coefficient (r) was calculated to explore potential predictors of balance performance within each group, while Spearman’s rho (*ρ*) was used for non-parametric variables. Correlation coefficients were interpreted based on Hopkins’ (2006) guidelines, as follows: 0.0–0.09 as trivial, 0.1–0.29 as small, 0.3–0.49 as moderate, 0.5–0.69 as large, 0.7–0.89 as very large, and 0.9–0.99 as nearly perfect. To examine differences in correlations between the two groups, Fisher’s z transformation was applied for comparison, using the Psychometrica calculator (https://www.psychometrica.de/correlation.html, accessed on 2 September 2024) [35].

Simple linear regression models were computed to identify the most robust strength parameters as predictors of the balance outcome variables, analyzed separately for each group. Assumptions for regression analysis included linearity of the relationships with balance tasks, a variance inflation factor < 5, absence of autocorrelation, homoscedasticity, and normality of residuals.

## 3. Results

The descriptive data of the groups showed significant statistical differences in all their characteristics (Table 1).

Strength and balance variables showed differences in practically all parameters, with better performance in the post-PHV group (Table 2), except in CMJ_Height_ (*p* = 0.083) and TTS (*p* = 0.926).

The primary morphological characteristics of the gymnasts (i.e., height and body mass) were indirectly associated with CoP excursion across both groups (Table 3). Overall, the circa-PHV group exhibited stronger associations than the post-PHV group, with significant differences observed in body mass (circa-PHV −0.975 ≥ r/*ρ* ≤ −0.863, post-PHV −0.806 ≥ r/*ρ* ≤ −0.297; 0.002 ≥ *p* ≤ 0.214) and body height (circa-PHV −0.940 ≥ r/*ρ* ≤ −0.741, post-PHV −0.727 ≥ r/*ρ* ≤ −0.415, 0.012 ≥ *p* ≤ 0.115). Body height was the only morphological variable significantly associated with strength variables, showing stronger positive correlations in the circa-PHV group (circa-PHV 0.920 ≥ r/*ρ* ≤ 0.524, post-PHV −0.236 ≥ r/*ρ* ≤ 0.643, 0.014 ≥ *p* ≤ 0.057).

Experience (not included in Table 3) was not significantly correlated with balance (circa-PHV, −0.379 ≥ r/*ρ* ≤ 0.034, 0.134 ≥ *p* ≤ 0.900; post-PHV, −0.494 ≥ r/*ρ* ≤ −0.092, 0.121 ≥ *p* ≤ 0.725) or strength variables (circa-PHV, 0.265 ≥ r/*ρ*≤ 0.487, 0.056 ≥ *p* ≤ 0.305; post-PHV, 0.144 ≥ r/*ρ* ≤ 0.367, 0.162 ≥ *p* ≤ 0.582), with the sole exception of CMJ_Height_ at post-PHV (r = 0.618, *p* = 0.008).

Correlational analysis revealed numerous significant associations between strength and balance measures (Table 4). The results showed negative associations between isometric strength tests (IMTP, −682 ≥ r/*ρ* ≤ −0.524, large, in circa-PHV, −578 ≥ r/*ρ* ≤ −0.306, moderate to large, in post-PHV; handgrip, −0.884 ≥ r/*ρ* ≤ −0.532, large to very large, in circa-PHV, −716 ≥ r/*ρ* ≤ −0.319, moderate to very large, in post-PHV) and CMJ_Power_ (−0.949 ≥ r/*ρ* ≤ −0.739, very large to nearly perfect, in circa-PHV, −745 ≥ r/*ρ* ≤ −0.136, small to very large, in post-PHV) with all balance measures (CoP excursion and TTS) in both the circa-PHV and post-PHV groups. Correlation coefficients were generally lower for the CMJ_Height_ and push-up tests, with positive values also appearing (CMJ_Height_, −0.567 ≥ r/*ρ* ≤ −0.187 in circa-PHV, small to large, 0.354 ≥ r/*ρ* ≤ −0.005, trivial to moderate, in post-PHV; push-ups, −0.637 ≥ r/*ρ* ≤ −0.393, moderate to large, in circa-PHV, −0.239 ≥ r/*ρ* ≤ 0.057, trivial to small, in post-PHV). The TTS only found significant associations with the handgrip in post-PHV (*ρ* = −0.559, large, *p* = 0.027) and with the CMJ in the circa-PHV (height, r = −0.539, large, *p* = 0.031; power, r = −0.674, large, *p* = 0.004).

Overall, more significant associations appeared in the circa-PHV group, also with higher coefficients. Specifically, when comparing the associations between both groups, a greater number of significant differences were observed (Table 4) in CMJ_Power_ with seven in total (with bilateral AP *p* = 0.006, and SP *p* = 0.011, tandem ML *p* = 0.02, SP *p* = 0.018, headstand AP *p* = 0.025, ML *p* = 0.028, SP *p* = 0.01), three in CMJ_Height_ (bilateral SP *p* = 0.049, tandem ML *p* = 0.017, SP *p* = 0.019), and one in both push-ups (headstand ML *p* = 0.046) and handgrip (bilateral AP *p* = 0.037). TTS showed no differences in any of the strength parameters, as did IMTP with any of the balance parameters.

Regression analysis revealed that push-ups did not predict any balance measure, and CMJ_Height_ only showed a significant regression with the Tandem_AP_ position within the circa-PHV group. Table 5 presents the strength measures that significantly predicted performance in the assessed balance parameters. Overall, only IMTP, handgrip, and CMJ_Power_ demonstrated predictive capacity across both maturational groups for bilateral on blocks and tandem static balance. In each case, a greater proportion of variance was explained in the circa-PHV group (0.894 ≥ R^2^ ≤ 0.427) compared to the post-PHV group (0.526 ≥ R^2^ ≤ 0.218). CMJ_Power_ emerged as the strength measure most predictive of balance parameters in both groups, followed by handgrip, with the tandem stance showing the highest association with strength variables among all tasks analyzed.

## 4. Discussion

In this study, the aim was to examine the relationship between specific strength capacities (power, muscular endurance, and maximal isometric strength) and various balance tasks, including static standing and inverted balance as well as landing stability, in circa-PHV and post-PHV female gymnasts. Findings revealed numerous significant associations between isometric strength, CMJ_Power_, and all balance measures, with stronger correlations noted in the circa-PHV group. CMJ power and handgrip emerged as the most predictive strength measures for balance performance across both groups, particularly in the static standing tasks performed on blocks and in tandem stance. Predictive capacity was consistently greater in the circa-PHV group, underscoring the influence of maturational stage on strength–balance interactions.

The gymnasts studied were separated by their maturation state, close or far from the age of the PHV. Circa-PHV gymnasts showed lower chronological age, weight, and sporting experience. They also exhibited lower performance in strength [36] and static balance (greater CoP excursion) [6,13,15] compared to the post-PHV. These results are in line with the ages and maturity status of both groups, suggesting greater stability and postural control in young individuals with a higher level of maturation [14], as well as a greater strength capacity [36]. In addition, morphological changes had a greater impact on static balance parameters in the circa-PHV group, with body mass and height showing significantly higher associations than in the post-PHV group. These findings suggest that the rapid increases in body weight and height typical of circa-PHV gymnasts [37] more markedly affect static task performance (e.g., standing and inverted balance) compared to the more gradual changes seen in post-PHV gymnasts. In terms of strength variables, body height was the only morphological factor that significantly differentiated the relationships between groups, further highlighting the stronger influence of morphological changes on strength in the circa-PHV group compared to the post-PHV group [38]. This pattern also aligns with the findings reported by Gebel et al. [24], observing that the young athletes with better balance achieved higher performance in physical tests.

Regarding the proactive balance test, the prior research suggests that jumping experience across sports (involving both male and female athletes) may improve coordination for post-landing stabilization [11]. Although coordination parameters were not assessed in this study, female gymnasts from both maturational groups displayed similar TTS values despite variations in sports experience. Furthermore, significant associations between body mass and height with TTS were found only in the circa-PHV group, indicating that post-landing stability at this stage may be predominantly influenced by morphological changes [39], with additional factors likely contributing at later stages. Further research with larger sample sizes, analyzing males and females separately across various age groups, is required to elucidate the impact of maturation on post-landing stabilization.

The analysis of associations between strength parameters and CoP excursion revealed the expected inverse relationships across static and proactive balance tasks in both gymnast groups [20,24], confirming our initial hypothesis with one exception, the positive but low (trivial to moderate) associations in CMJ_Height_ were observed in the post-PHV group. Overall, these findings underscore the importance of high maximal strength, power, and muscular endurance levels for enhancing stability in young gymnasts, across static standing and inverted tasks as well as for post-landing stability.

Specifically, the strongest associations, with correlations from large to nearly perfect, were found between CMJ_Power_ and static standing tasks, also with TTS but with slightly weaker correlations. These results are logical given that CMJ_Power_ calculations incorporate body mass [31] and are consistent with evidence linking growth (increasing body mass) to reduced CoP excursion [6,13]. Furthermore, strength and power have been reported to more strongly affect balance in children and young adolescents than in adults [40]. Just as a more relevant role of CMJ_Power_ has been observed in static standing tasks, both tasks with a primary action of the lower body, large correlations were also found between the handgrip and headstand stability, since both tasks mainly involve the upper body with a marked support of the hands on the ground to maintain balance [6]. In contrast, the lower associations found between push-ups and headstand tests may reflect task dissimilarity; while the push-up test assesses upper-body muscular endurance through repetitive motion to failure, headstands require sustained isometric contraction without muscular fatigue. This weak link between endurance training and balance has also been reported in adults [41].

Analyzing balance tasks, the tandem position was most sensitive to strength parameters in both groups, likely due to its rarity and the narrow base of support, which makes it more challenging and discriminative in our subjects [42]. Regarding proactive balance, the results generally showed weaker associations with strength tests than static balance, indicating distinct balance behaviors across task types. Collectively, these findings reinforce the concept that balance is task-specific [4,31], with each type and each balance task associated with different physical capacities.

Previous studies with children from other age groups prior to the circa-PHV [20,21,22,23], have shown weak to non-existent associations regarding the relationship between strength and balance. Granacher and Muehlbauer [23] reported reductions in CoP excursion, as well as improvements in CMJ_Height_, maximal torque, and RFD in the plantar flexors after a balance training intervention. However, despite these improvements, no statistically significant correlations were found between the balance and strength tests. Granacher and Gollhofer [21] further supported these findings, reporting no relationship between similar strength variables with CoP excursion in static or dynamic tasks. Similarly, Muehlbauer et al. [22] did not observe significant associations between maximal isometric leg press strength, CMJ height, with static or proactive balance (the Functional Reach Test). Hammami et al. [20] reported significant correlations in the pre-PHV group between strength and power measures with balance proxies, although less frequent and lower when compared to the circa-PHV and post-PHV groups.

To understand these divergences, it is noteworthy that some of these studies involved samples mixing non-athletic boys and girls aged between 6 and 10 years [21,22,23]. The immaturity of the postural control system at these ages may explain these findings [20,23], and better coordination in the athletic population would contribute to better balance performance compared to non-athletic boys [20]. Furthermore, although these ages are well below PHV and therefore differ considerably from the sample in our study, our sample consisted exclusively of female participants, whose typical pubertal onset occurs before boys, between the ages of 8 and 13 [43].

Additionally, these studies employed the traditional bipedal stance as a steady-state static balance task, which may lack the discriminatory sensitivity needed for analyzing balance in healthy subjects [30,31]. Muehlbauer, Gollhofer et al. [44] investigated an adult sample of 18 males and 19 females, all of an average age of 23, and similarly found no correlations between static or dynamic balance and isometric strength or power. It is notable that these participants, significantly older than our sample, had completed their growth and maturation processes, likely resulting in a reduced influence of strength on balance [40]. In addition, these adults lacked specific training in strength, power, or balance [44], and the mixed-sex sample may have introduced variability in load–velocity profiles and force levels [45], potentially affecting the responses to balance tasks.

The second hypothesis was partially supported, as significant correlations between strength and power indicators with different balance tasks were more frequent and higher in the circa-PHV group. However, differences in correlation coefficients between the circa- and post-PHV groups depended on the type of strength measured. Our findings align with those of Hammami et al. [20], who observed relationships between static (Stork test) and dynamic (Y test) balance performance and back extensor isometric strength in young male soccer players. These associations were stronger in the circa-PHV group than in the post-PHV group, though without significant group differences [20]. In a similar pattern, the associations we found between overall maximal isometric strength (e.g., IMTP) and localized strength (e.g., handgrip) with balance among young female gymnasts showed more consistency across maturational stages, though trends toward statistical significance were noted for handgrip (*p* ≈ 0.1). These results underscore a higher importance of maximal isometric strength in balance performance, regardless of maturational status in young gymnasts.

Hammami et al. [20] also observed stronger correlations between various jump tests and balance tests in the circa-PHV group than in the post-PHV group, though without significant differences. However, in our study, numerous significant differences (*p* < 0.05) and trends toward significance (*p* < 0.1) were found between CMJ height and power and both static and proactive balance. This finding suggests a greater influence of jumping ability on balance in circa-PHV athletes, likely due to variations in body growth (Table 3) and neuromuscular development, which are particularly impactful on motor control around PHV ages [20,40]. This argument may also explain the different relationships observed between muscular endurance (i.e., push-up test) and balance measures when comparing groups, with a greater influence seen in circa-PHV athletes.

The third hypothesis was confirmed. Although the number of balance variables predicted by strength measures was similar across both maturational groups, the variance explained was consistently higher in the circa-PHV group. Specifically, strength measures predicting balance across both groups explained between 42.7% and 89.4% of the variance in the circa-PHV group compared to 21.8% to 52.6% in post-PHV. This greater predictive capacity in the circa-PHV group underscores the role of biological development and neuromuscular maturation in shaping the relationship between maximal isometric strength, muscle power, and static balance performance, aligning with the review of [40].

Another possible reason for the stronger influence of strength on balance in younger athletes could be attributed to differences in task automation. During initial stages of practice, motor control tends to be less specific; however, as experience increases, so does movement automation, leading to more refined motor control and improved performance [40]. In the present study, no significant associations were found between experience and the strength or balance variables analyzed, suggesting that maturational state may have a stronger impact on balance performance than experience alone.

The predictive importance of maximal isometric strength and CMJ_Power_ on static balance performance was further emphasized, while muscular endurance and vertical jump height showed lower predictive relevance in both circa- and post-PHV groups. These results suggest that strength training could enhance postural performance, although the impact appears to vary by maturational stage and the specificity of the training [40,46]. Thus, strength training might be more beneficial for improving balance in individuals with incomplete strength development, as seen in younger populations [20,40,47], or in older adults facing strength declines [40,46].

Recognizing that postural performance can be evaluated through specific sport-related tasks versus more generalized tasks [31], in this study, both task types were included. Here, the least sport-specific tasks were the standing balance tasks, with tandem stance being most strongly predicted by strength measures. Although this non-ecological task (i.e., non-sport-specific) may have appeared challenging and novel for the gymnasts [31], the findings underscore the predictive role of strength. Paillard [31] suggested that ecological, or sport-specific, postural tasks are better suited for studying the relationship between sports and postural performance. The headstand, a commonly trained and specific skill among the gymnasts, was mainly predicted by handgrip isometric strength and CMJ power in the post-PHV group, explaining only 24.9% and 22.9% of the variance, respectively. Similarly, for proactive balance, time to stabilize after a landing was only predicted by CMJ power (41.6% of variance explained) in circa-PHV. In this sense, the future research should aim to identify strength variables that better explain performance in specific postural tasks relevant to acrobatic gymnastics, regardless of the athlete’s maturational stage.

Based on the findings of this study, training for adolescent gymnasts should incorporate, in addition to classic dynamic strength exercises, targeted work on power and maximal isometric strength. Technique should always be prioritized over intensity, with exercises carefully adapted to the individual’s capacity and maturational stage (especially in circa-PHV). For isometric strength, incorporating static standing and inverted positions with appropriately weights of short duration is recommended. To develop specific power, exercises such as maximal horizontal and vertical jumps performed with maximum velocity, as well as upper-body horizontal and vertical pushing movements (e.g., medicine ball throws), could be included.

Finally, it should be noted that these findings must be interpreted with caution due to several limitations in this research. First, the cross-sectional design restricts the possibility of establishing definitive cause-and-effect relationships between strength and balance. This sample consisted solely of female acrobatic gymnasts, for whom static maintenance is a primary performance factor; thus, results may not fully generalize to young athletes in other sports where different strength–balance dynamics could be at play. Additionally, the sample size was limited by the availability of participants meeting the study criteria, potentially reducing the statistical power of the results.

## 5. Conclusions

This study is the first in which associations between different strength and balance indicators were examined according to maturational stages in young acrobatic gymnasts. The findings demonstrate a clear relationship, particularly between maximal isometric strength and CMJ power with static steady-state balance (standing and inverted) in both circa-PHV and post-PHV gymnasts. Strength’s influence on balance was notably stronger in the circa-PHV group, underscoring the pivotal role of maturational stage in strength–balance dynamics, with morphological changes associated with PHV affecting both static and proactive balance tasks.

Associations between maximal isometric strength and balance were found to be relatively consistent across both maturation stages, with minimal significant variations, underscoring the critical role of this strength attribute in balance performance regardless of the athlete’s stage of maturation. This contrasts with jumping ability, where greater differences between maturation stages were observed.

The task-specific nature of balance was also highlighted, as distinct balance types (static, proactive) and tasks (standing, headstand) demonstrated varying levels of correlation with different strength indicators. These findings suggest that certain balance tasks may require specific physical capacities depending on the athlete’s maturation stage.

Given that gymnasts in the same competitive category can vary in maturation, these findings suggest that training loads and strength demands for balance tasks should be tailored according to the athlete’s proximity to PHV. With consideration of this study’s limitations, this research provides a foundation for designing strength training interventions aimed at enhancing balance in young gymnasts. The implications extend to optimizing performance in sports that demand high levels of postural control.

## Figures and Tables

**Table 1:** Descriptive characteristics of the gymnasts by group.

Characteristics	(−2.2. ≥ PHV ≤ 2.09) Circa-PHV	(2.1 ≥ PHV ≤ 5.6) Post-PHV
	Mean ± SD	Mean ± SD
Age ^†^ (years)	11.92 ± 1.67	16.47 ± 1.80 ***
Maturity offset ^†^	−0.29 ± 1.50	3.48 ± 1.40 ***
Body mass (kg)	38.46 ± 9.31	54.05 ± 7.52 ***
Body height (cm)	146.13 ± 10.28	160.79 ± 5.76 ***
Body mass index (kg/m^2^)	17.72 ± 2.65	20.84 ± 2.13 ***
Experience (years)	6.05 ± 2.19	8.82 ± 2.21 ***

Peak height velocity (PHV). ^†^ Mann–Whitney test. *** *p* < 0.001.

**Table 2:** Strength, static, and proactive balance measurements according to peak height velocity.

			Circa-PHV	Post-PHV
Variables			Mean ± SD	Mean ± SD
Strength		IMTP (N)	917.88 ± 256.52	1433.56 ± 292.73 ***
		Handgrip (kg)	21.98 ± 5.50	31.04 ± 5.09 ***
		CMJ_Height_ (cm)	27.76 ± 3.87	29.89 ± 3.01
		CMJ_Power_ (W)	1314.91 ± 568.00	2187.98 ± 383.22 ***
		Push-Ups (n)	16.33 ± 7.78	^†^ 21.37 ± 8.27 *
Static balance ^♦^	Bilateral	AP (mm)	1.72 ± 0.50	1.21 ± 0.16 ***
		ML (mm)	2.23 ± 0.77	^†^ 1.60 ± 0.46 **
		SP (mm/s)	0.12 ± 0.03	0.08 ± 0.01 ***
	Headstand	AP (mm)	0.75 ± 0.20	0.50 ± 0.07 ***
		ML (mm)	0.89 ± 0.22	0.65 ± 0.08 ***
		SP (mm/s)	0.18 ± 0.04	0.13 ± 0.01 ***
	Tandem	AP (mm)	1.92 ± 0.58	1.25 ± 0.17 ***
		ML (mm)	2.66 ± 0.77	1.74 ± 0.23 ***
		SP (mm/s)	0.13 ± 0.04	0.09 ± 0.01 ***
Proactive balance		TTS (s)	0.56 ± 0.15	^†^ 0.57 ± 0.18

Isometric mid-thigh pull test (IMTP). ^♦^ Centre of pressure parameters normalized by height: anteroposterior (AP), mediolateral (ML), speed (SP). Time to stabilization (TTS) after landing. Peak height velocity (PHV). ^†^ Mann–Whitney test. * *p* < 0.05, ** *p* < 0.01, *** *p* < 0.001.

**Table 3:** Correlation coefficients between morphological characteristics and balance measures studied in circa-PHV and post-PHV. Differences between the associations of both groups.

			Body Mass			Body Height			BMI		
			Circa-PHV	Post-PHV	*p*	Circa-PHV	Post-PHV	*p*	Circa-PHV	Post-PHV	*p*
Static balance	Bilateral	AP	^†^ −0.946 ***	−0.605 *	*0.002*	−0.894 ***	−0.606 **	*0.025*	−0.864 ***	−0.420	*0.011*
		ML	^†^ −0.863 ***	^†^ −0.764 ***	0.214	^†^ −0.775 ***	−0.424	0.062	^†^ −0.770 ***	−0.548 *	0.142
		SP	^†^ −0.975 ***	−0.806 ***	*0.002*	−0.940 ***	−0.709 **	*0.012*	−0.842 ***	−0.627 **	0.097
	Tandem	AP	−0.869 ***	−0.688 **	0.1	−0.891 ***	−0.568 *	*0.019*	^†^ −0.760 ***	−0.562 *	0.17
		ML	^†^ −0.958 ***	−0.795 ***	*0.013*	−0.913 ***	−0.727 ***	0.05	−0.790 ***	−0.602 *	0.16
		SP	−0.905 ***	−0.772 ***	0.105	−0.906 ***	−0.666 **	*0.032*	−0.786 ***	−0.612 **	0.178
	Headstand	AP	−0.886 ***	−0.437	*0.009*	−0.741 ***	−0.447	0.115	−0.903 ***	−0.289	*0.001*
		ML	^†^ −0.946 ***	−0.633 *	*0.004*	−0.886 ***	−0.607 *	*0.038*	^†^ −0.868 ***	−0.439	*0.015*
		SP	−0.885 ***	−0.297	*0.003*	−0.750 ***	−0.415	0.088	−0.888 ***	−0.122	*0.001*
Proactive balance		TTS	−0.607 *	−0.067	0.052	−0.506 *	−0.094	0.119	−0.658 **	−0.036	*0.027*
Strength		IMTP	0.726 **	^†^ 0.636 **	0.331	^†^ 0.756 ***	^†^ 0.362	0.057	0.532 *	^†^ 0.530 *	0.497
		Handgrip	0.875 ***	^†^ 0.717 **	0.12	0.834 ***	0.473	*0.037*	0.749 ***	0.681 **	0.358
		CMJ_Height_	0.410	−0.113	0.073	0.524 *	−0.180	*0.022*	0.275	−0.022	0.21
		CMJ_Power_	0.947 ***	0.914 ***	0.254	0.920 ***	0.643 **	*0.014*	0.830 ***	^†^ 0.844 ***	0.451
		Push-Ups	^†^ 0.597 *	^†^ 0.080	0.054	^†^ 0.456	−0.236	*0.034*	0.425	^†^ 0.268	0.327

Peak height velocity (PHV). isometric mid-thigh pull test (IMTP). Anteroposterior (AP), mediolateral (ML), speed (SP). time to stabilization (TTS) after landing. Body mass index (BMI). Significant correlations with * *p* < 0.05, ** *p* < 0.01, *** *p* < 0.001. ^†^ Spearman’s correlations. *Italic* in *p* < 0.05 comparing groups.

**Table 4:** Correlation coefficients between strength and balance measures studied in circa-PHV and post-PHV. Differences between the associations of both groups.

			IMTP			Handgrip			CMJ_Height_			CMJ_Power_			Push-Ups		
			Circa-PHV	Post-PHV	*p*	Circa-PHV	Post-PHV	*p*	Circa-PHV	Post-PHV	*p*	Circa-PHV	Post-PHV	*p*	Circa-PHV	Post-PHV	*p*
Static balance	Bilateral	AP	−0.647 **	−0.376	0.165	−0.801 ***	−0.391	*0.037*	−0.439	0.092	0.068	−0.914 ***	−0.543 *	*0.006*	^†^ −0.491	^†^ 0.105	0.054
		ML	−0.467	^†^ −0.578 *	0.345	−0.532 *	^†^ −0.593 *	0.408	−0.307	^†^ 0.001	0.201	−0.739 ***	^†^ −0.699 **	0.413	^†^ −0.411	^†^ −0.027	0.153
		SP	−0.682 **	−0.516 *	0.248	−0.884 ***	−0.676 **	0.069	−0.505 *	0.071	*0.049*	−0.949 ***	−0.745 ***	*0.011*	^†^ −0.547 *	^†^ −0.031	0.073
	Tandem	AP	−0.669 **	−0.401	0.159	−0.815 ***	−0.603 *	0.125	−0.507 *	−0.005	0.071	−0.876 ***	−0.663 **	0.077	^†^ −0.610 *	^†^ −0.134	0.076
		ML	−0.649 **	−0.306	0.117	−0.817 ***	−0.582 *	0.105	−0.549 *	0.186	*0.017*	−0.924 ***	−0.687 **	*0.02*	^†^ −0.637 *	^†^ −0.239	0.102
		SP	−0.679 **	−0.327	0.103	−0.842 ***	−0.631 **	0.104	−0.567 *	0.142	*0.019*	−0.926 ***	−0.683 **	*0.018*	^†^ −0.615 *	^†^ −0.236	0.117
	Headstand	AP	−0.553 *	−0.276	0.198	−0.769 ***	−0.630 *	0.245	−0.187	0.354	0.078	−0.776 ***	−0.261	*0.025*	^†^ −0.450	^†^ −0.120	0.186
		ML	−0.578 *	−0.389	0.267	−0.779 ***	−0.716 **	0.36	−0.423	0.152	0.062	−0.873 ***	−0.533 *	*0.028*	^†^ −0.565 *	^†^ 0.048	*0.046*
		SP	−0.524 *	−0.330	0.275	−0.787 ***	−0.550 *	0.133	−0.204	0.335	0.079	−0.782 ***	−0.136	*0.01*	^†^ −0.393	^†^ 0.057	0.124
Proactive balance		TTS	−0.319	^†^ −0.424	0.38	−0.480	^†^ −0.559 *	0.393	−0.539 *	^†^ 0.088	*0.034*	−0.674 **	^†^ −0.247	0.75	^†^ −0.496	^†^ −0.179	0.192

Peak height velocity (PHV). Isometric mid-thigh pull test (IMTP). Anteroposterior (AP), mediolateral (ML), speed (SP). Time to stabilization (TTS) after landing. Significant correlations with * *p* < 0.05, ** *p* < 0.01, *** *p* < 0.001. ^†^ Spearman’s correlations. *Italic* in *p* < 0.05 comparing groups.

**Table 5:** Linear regression analysis with measures of balance and strength as dependent and independent variables, respectively, circa- and post-PHV groups.

Predictor Variables		Predicted Variables											
		Static Balance ^♦^											Proactive Balance
		Bilateral			Tandem						Headstand		
		AP	SP		AP		ML		SP		ML	SP	TTS
		Post-PHV	Circa-PHV	Post-PHV	Circa-PHV	Post-PHV	Circa-PHV	Post-PHV	Circa-PHV	Post-PHV	Post-PHV	Post-PHV	Circa-PHV
IMTP	R^2^adj	N.I.	*0.427*	*0.218*	0.408	N.I.	0.380	N.I.	0.423	N.I.	N.I.	N.I.	N.I.
	F		*12.16*	*5.45*	11.33		10.18		11.99				
	*p*		*0.004*	*0.034*	0.005		0.007		0.004				
Handgrip	R^2^adj	N.I.	N.I.	N.I.	*0.641*	*0.321*	*0.644*	*0.294*	*0.688*	*0.357*	N.I.	0.249	N.I.
	F				*27.75*	*8.56*	*28.18*	*7.68*	*34.06*	*9.89*		5.63	
	*p*				*<0.001*	*0.01*	*<0.001*	*0.014*	*<0.001*	*0.007*		0.034	
CMJ_Height_	R^2^adj	N.I.	N.I.	N.I.	0.208	N.I.	N.I.	N.I.	N.I.	N.I.	N.I.	N.I.	N.I.
	F				5.201								
	*p*				0.038								
CMJ_Power_	R^2^adj	0.248	*0.894*	*0.526*	*0.753*	*0.402*	*0.843*	*0.437*	*0.848*	*0.431*	0.229	N.I.	0.416
	F	6.29	*135.30*	*1.87*	*49.66*	*11.74*	*86.95*	*13.41*	*90.55*	*13.13*	5.17		11.70
	*p*	0.024	*<0.001*	*<0.001*	*<0.001*	*0.004*	*<0.001*	*0.002*	*<0.001*	*0.002*	0.041		0.004

^♦^ Centre of pressure excursion normalized by height: anteroposterior (AP), mediolateral (ML), speed (SP). Isometric mid-thigh pull test (IMTP). Time to stabilization (TTS). Peak height velocity (PHV). R^2^ adjusted (R^2^adj). *Italic* in parameters that predict in both maturity groups. Not included in the regression model (N.I.).

## Data Availability

Data cannot be shared publicly because it is confidential. The data pertains to humans, including minors. Data access requests can be made to L. Arturo Gómez-Landero, from the Universidad Pablo de Olavide. Email: lagomrod@upo.es.

## References

[1] Bull F.C., Al-Ansari S.S., Biddle S., Borodulin K., Buman M.P., Cardon G., Carty C., Chastin S., Colley R.C., Dempsey P.C. (2020). World Health Organization 2020 guidelines on physical activity and sedentary behaviour. Br. J. Sports Med..

[2] Hrysomallis C. (2011). Balance ability and athletic performance. Sports Med..

[3] Federation Internationale de Gymnastique (2024). Code of Points 2025–2028. Acrobatic Gymnastics.

[4] Shumway-Cook A., Woollacott M.H. (2016). Motor Control: Translating Research Into Clinical Practice.

[5] Paillard T., Noé F. (2015). Techniques and methods for testing the postural function in healthy and pathological subjects. BioMed Res. Int..

[6] Gómez-Landero L.A., Leal del Ojo P., Walker C., Floría P. (2021). Static balance performance differs depending on the test, age and specific role played in acrobatic gymnastics. Gait Posture.

[7] Floría P., Gómez-Landero L.A., Harrison A.J. (2015). Centre of pressure correlates with pyramid performance in acrobatic gymnastics. Sports Biomech..

[8] Leal Del Ojo P., Floría P., Harrison A.J., Gómez-Landero L.A. (2020). Effects of task difficulty on centre of pressure excursion and its inter-trial variability in acrobatic gymnastics pyramid performance. Sports Biomech..

[9] Byrne A., Lodge C., Wallace J. (2021). Test–retest reliability of single-leg time to stabilization following a drop-landing task in healthy individuals. J. Sport Rehabil..

[10] Ringhof S., Stein T. (2018). Biomechanical assessment of dynamic balance: Specificity of different balance tests. Hum. Mov. Sci..

[11] Monfort-Torres G., García-Massó X., Skýpala J., Blaschová D., Estevan I. (2024). Coordination and coordination variability during single-leg drop jump landing in children. Hum. Mov. Sci..

[12] Federation Internationale de Gymnastique (2024). 2025–2028 Youth & Juniors Rules. Acrobatic Gymnastics.

[13] Verbecque E., Vereeck L., Hallemans A. (2016). Postural sway in children: A literature review. Gait Posture.

[14] John C., Rahlf A.L., Hamacher D., Zech A. (2019). Influence of biological maturity on static and dynamic postural control among male youth soccer players. Gait Posture.

[15] Gómez-Landero L.A., Leal Del Ojo P., Floría P. (2024). Analysis of proactive, static and dynamic unipedal balance in young gymnasts during adolescence. ISBS Proc. Arch..

[16] Holden S., Boreham C., Delahunt E. (2016). Sex differences in landing biomechanics and postural stability during adolescence: A systematic review with meta-analyses. Sports Med..

[17] Lehnert M., Krejčí J., Janura M., De Ste Croix M. (2022). Age-related changes in landing mechanics in elite male youth soccer players: A longitudinal study. Appl. Sci..

[18] Wren T.A.L., O’Callahan B., Katzel M.J., Zaslow T.L., Edison B.R., VandenBerg C.D., Conrad-Forrest A., Mueske N.M. (2020). Movement variability in pre-teen and teenage athletes performing sports-related tasks. Gait Posture.

[19] Lesinski M., Schmelcher A., Herz M., Puta C., Gabriel H., Arampatzis A., Behringer M., Gollhofer A. (2020). Maturation-, age-, and sex-specific anthropometric and physical fitness percentiles of German elite young athletes. PLoS ONE.

[20] Hammami R., Chaouachi A., Makhlouf I., Granacher U., Behm D.G. (2016). Associations between balance and muscle strength, power performance in male youth athletes of different maturity status. Pediatr. Exerc. Sci..

[21] Granacher U., Gollhofer A. (2012). Is there an association between variables of postural control and strength in prepubertal children?. J. Strength Cond. Res..

[22] Muehlbauer T., Besemer C., Wehrle A., Gollhofer A., Granacher U. (2013). Relationship between strength, balance and mobility in children aged 7–10 years. Gait Posture.

[23] Granacher U., Muehlbauer T., Maestrini L., Zahner L., Gollhofer A. (2011). Can balance training promote balance and strength in prepubertal children?. J. Strength Cond. Res..

[24] Gebel A., Prieske O., Behm D.G., Granacher U. (2020). Effects of balance training on physical fitness in youth and young athletes: A narrative review. Strength Cond. J..

[25] Federation Internationale de Gymnastique (2024). Code of Points Acrobatic Gymnastics. Tables of Difficulty 2025–2028.

[26] De Miguel-Etayo P., Gracia-Marco L., Ortega F.B., Intemann T., Foraita R., Lissner L., Moreno L.A., Sjöström M., Veidebaum T., Ahrens W. (2014). Physical fitness reference standards in European children: The IDEFICS study. Int. J. Obes..

[27] Moore S.A., McKay H.A., Macdonald H., Nettlefold L., Baxter-Jones A.D.G., Cameron N., Olivier M., Mirwald R.L., Bailey D.A., Baugh A. (2015). Enhancing a somatic maturity prediction model. Med. Sci. Sports Exerc..

[28] Comfort P., Dos’Santos T., Beckham G.K., Stone M.H., Guppy S.N., Haff G.G. (2019). Standardization and methodological considerations for the isometric midthigh pull. Strength Cond. J..

[29] Baumgartner T.A., Oh S., Chung H., Hales D. (2002). Objectivity, reliability, and validity for a revised push-up test protocol. Meas. Phys. Educ. Exerc. Sci..

[30] Kiers H., Van Dieën J., Dekkers H., Wittink H., Vanhees L. (2013). A systematic review of the relationship between physical activities in sports or daily life and postural sway in upright stance. Sports Med..

[31] Paillard T. (2019). Relationship between sport expertise and postural skills. Front. Psychol..

[32] Sayers S.P., Harackiewicz D.V., Harman E.A., Frykman P.N., Rosenstein M.T. (1999). Cross-validation of three jump power equations. Med. Sci. Sports Exerc..

[33] Agostini V., Chiaramello E., Canavese L., Bredariol C., Knaflitz M. (2013). Postural sway in volleyball players. Hum. Mov. Sci..

[34] Liljequist D., Elfving B., Skavberg Roaldsen K. (2019). Intraclass correlation—A discussion and demonstration of basic features. PLoS ONE.

[35] Diedenhofen B., Musch J. (2015). cocor: A comprehensive solution for the statistical comparison of correlations. PLoS ONE.

[36] Castro-Piñero J., González-Montesinos J.L., Mora J., Keating X.D., Girela-Rejón M.J., Sjöström M., Ruiz J.R. (2009). Percentile values for muscular strength field tests in children aged 6 to 17 years: Influence of weight status. J. Strength Cond. Res..

[37] Malina R.M. (1994). Physical growth and biological maturation of young athletes. Exerc. Sport Sci. Rev..

[38] Roemmich J.N., Rogol A.D. (1995). Physiology of growth and development. Clin. Sports Med..

[39] Rhodes D., Maden-Wilkinson J., Jeffrey J., Birdsall D., Alexander J. (2020). Measures of PHV and the effect on directional dynamic stability to identify risk factors for injury in elite football. J. Sports Med. Phys. Fitness.

[40] Muehlbauer T., Gollhofer A., Granacher U. (2015). Associations between measures of balance and lower-extremity muscle strength/power in healthy individuals across the lifespan: A systematic review and meta-analysis. Sports Med..

[41] Kloubec J.A. (2010). Pilates for improvement of muscle endurance, flexibility, balance, and posture. J. Strength Cond. Res..

[42] Muehlbauer T., Roth R., Bopp M., Granacher U. (2012). An exercise sequence for progression in balance training. J. Strength Cond. Res..

[43] Lee J.M., Appugliese D., Kaciroti N., Corwyn R.F., Bradley R.H., Lumeng J.C. (2007). Weight status in young girls and the onset of puberty. Pediatrics.

[44] Muehlbauer T., Gollhofer A., Granacher U. (2013). Association of balance, strength, and power measures in young adults. J. Strength Cond. Res..

[45] Torrejón A., Balsalobre-Fernández C., Haff G.G., García-Ramos A. (2019). The load-velocity profile differs more between men and women than between individuals with different strength levels. Sports Biomech..

[46] Paillard T. (2017). Plasticity of the postural function to sport and/or motor experience. Neurosci. Biobehav. Rev..

[47] Hammami R., Granacher U., Makhlouf I., Behm D.G., Chaouachi A. (2016). Sequencing effects of balance and plyometric training on physical performance in youth soccer athletes. J. Strength Cond. Res..

